# Chemical Communication of Antibiotic Resistance by a Highly Resistant Subpopulation of Bacterial Cells

**DOI:** 10.1371/journal.pone.0068874

**Published:** 2013-07-03

**Authors:** Omar M. El-Halfawy, Miguel A. Valvano

**Affiliations:** 1 Centre for Human Immunology and Department of Microbiology and Immunology, University of Western Ontario, London, Ontario, Canada; 2 Department of Pharmaceutical Microbiology, Faculty of Pharmacy, Alexandria University, Alexandria, Egypt; 3 Centre for Infection and Immunity, Queen's University Belfast, Belfast, United Kingdom; Imperial College London, United Kingdom

## Abstract

The overall antibiotic resistance of a bacterial population results from the combination of a wide range of susceptibilities displayed by subsets of bacterial cells. Bacterial heteroresistance to antibiotics has been documented for several opportunistic Gram-negative bacteria, but the mechanism of heteroresistance is unclear. We use *Burkholderia cenocepacia* as a model opportunistic bacterium to investigate the implications of heterogeneity in the response to the antimicrobial peptide polymyxin B (PmB) and also other bactericidal antibiotics. Here, we report that *B. cenocepacia* is heteroresistant to PmB. Population analysis profiling also identified *B. cenocepacia* subpopulations arising from a seemingly homogenous culture that are resistant to higher levels of polymyxin B than the rest of the cells in the culture, and can protect the more sensitive cells from killing, as well as sensitive bacteria from other species, such as *Pseudomonas aeruginosa* and *Escherichia coli*. Communication of resistance depended on upregulation of putrescine synthesis and YceI, a widely conserved low-molecular weight secreted protein. Deletion of genes for the synthesis of putrescine and YceI abrogate protection, while pharmacologic inhibition of putrescine synthesis reduced resistance to polymyxin B. Polyamines and YceI were also required for heteroresistance of *B. cenocepacia* to various bactericidal antibiotics. We propose that putrescine and YceI resemble "danger" infochemicals whose increased production by a bacterial subpopulation, becoming more resistant to bactericidal antibiotics, communicates higher level of resistance to more sensitive members of the population of the same or different species.

## Introduction

Treating infection caused by multidrug resistant bacteria is challenging, especially in immunocompromised patients. These individuals often succumb from infections by opportunistic bacteria that display intrinsic, high-level resistance to virtually all antimicrobials available for clinical use. Reduced permeability of the bacterial cell envelope in conjunction with multidrug efflux pumps are considered major determinants of intrinsic multidrug resistance [1]. However, the overall resistance of a bacterial population results from the combination of a wide range of susceptibilities displayed by subsets of bacterial cells. Bacterial heteroresistance to antibiotics has been documented for several pathogenic bacteria, but the mechanism of heteroresistance is not always clear. Here, we use *Burkholderia cenocepacia* as a model opportunistic bacterium to investigate the implications of heterogeneity in the response to the antimicrobial peptide polymyxin B (PmB) and also other antibiotics. *B. cenocepacia* is an environmental, opportunistic pathogen that causes serious infections in patients with cystic fibrosis and expresses high-level multidrug resistance [2]. Using the prototypic *B. cenocepacia* K56-2 strain, we observed a population-wide variation in the response to PmB and more importantly, that the more resistant members communicate higher level of resistance to less resistant members of the same population, and to other bacterial species in co-culture, such as *Pseudomonas aeruginosa* and *Escherichia coli*. Communication of increased resistance depended on overproduction by the more resistant subpopulations of the polyamine putrescine and increased secretion of YceI, a highly conserved small protein of unknown function. This rather general multifactorial mechanism of communication of antibiotic resistance is distinct from previously reported population-based resistance involving production of indole [3], [4], biogenic ammonia [5], and intercellular nanotubes [6]. Our findings uncover a novel, non-genetic and cooperative mechanism of transient increase in resistance that can be chemically communicated from more resistant members of a heterogeneous population to less resistant bacterial cells of the same or other species.

## Results and Discussion

### Heteroresistance of *B. cenocepacia* to PmB

**Figure 1:**
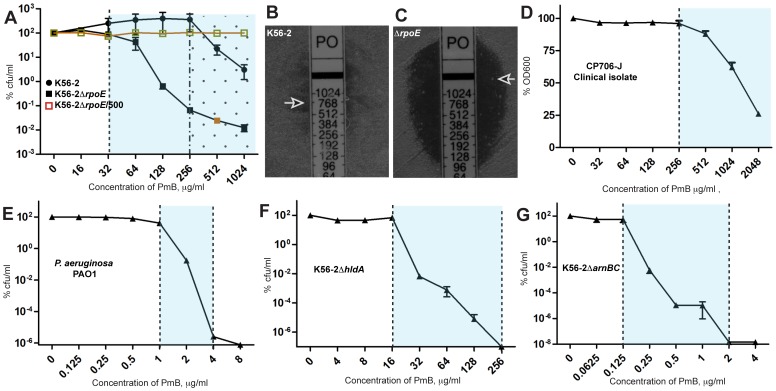
Heterogeneous response of *B. cenocepacia* to PmB. Population analysis profiling (PAP) of *B. cenocepacia* strains K56-2, K56-2Δ*rpoE*, K56-2Δ*rpoE*/500, CF706-J, K56-2Δ*hldA*, and K56-2Δ*arnBC*, and *P. aeruginosa* PAO1 by agar dilution at 24 h except for CF706-J by broth dilution at 18 h. n = 6. Panels (B) and (C), E-test showing discrete colonies at otherwise clear zones of inhibition, indicating heterogeneous response to PmB.

The prototypic *B. cenocepacia* clinical strain K56-2 was assessed for heteroresistance by performing population analysis profiling (PAP) of cultures exposed to serial dilutions of PmB. The percent growth inhibition increased gradually at high concentrations of PmB but without reaching complete bacterial inhibition, revealing residual subpopulations of more resistant cells (Figure 1A) and suggesting heteroresistance. However, the limited solubility of PmB in the culture medium at concentrations higher than 2,048 µg/ml precluded the determination of the exact minimal inhibitory concentration (MIC) for PmB against K56-2. To investigate this phenomenon in more detail, we performed PAP in isogenic mutants with intermediate sensitivity to PmB. K56-2Δ*rpoE*, which lacks an extracytoplasmic stress response regulator [7], showed evident heteroresistance to PmB (Figure 1A). A fraction of bacteria from the same culture was inhibited at 64 µg/ml despite that the MIC of PmB against the entire bacterial population was higher than 1,024 µg/ml. Gradual reduction in the resistant subpopulation was observed upon increasing PmB concentrations over a 16-fold range. Heteroresistance to PmB was confirmed by E-test, which demonstrated small colonies growing within the zone of inhibition surrounding the highest concentrations of PmB on the E-test strips, both in K56-2 and K56-2Δ*rpoE* (Figure 1B and C respectively). A similar pattern of heteroresistance was also previously observed for the K56-2Δ*suhB* [8], which lacks an inositol monophosphatase and like K56-2Δ*rpoE*, has intermediate sensitivity to PmB. Heteroresistance to PmB was also observed in the *B. cenocepacia* clinical isolate CP706-J, indicating that it is not a phenomenon unique to a single strain (Figure1D). In contrast, *P. aeruginosa* PAO1 did not show heteroresistance to PmB, as demonstrated by the abrupt drop in the bacterial growth on a two-fold increase of PmB concentration to reach complete growth inhibition (Figure 1E).

Heteroresistance to PmB was not related to the level of PmB resistance since mutants displaying high sensitivity to PmB were also heteroresistant. K56-2Δ*hldA*, a strain lacking the ability to produce a complete lipopolysaccharide (LPS) molecule as a result of the loss of the *hldA* gene [9], demonstrated heteroresistance to PmB at concentrations ranging from 32 µg/ml to 256 µg/ml (Figure 1F). Furthermore, K56-2Δ*arnBC* carrying a deletion of genes required for 4-amino-4-deoxy-l-arabinose (Ara4N) synthesis displayed similar heterogeneity in the response to PmB despite its exquisite sensitivity to PmB (Figure 1G). Since *B. cenocepacia* LPS modification with Ara4N is the major determinant for the extreme resistance to PmB [10] our results suggest that the heteroresistance of *B. cenocepacia* to PmB is not associated to LPS modifications and therefore depends on a different mechanism.

### A More Resistant Subpopulation of *B. cenocepacia* Protects Naïve Bacteria from PmB

We investigated whether the more resistant subpopulations of *B. cenocepacia* could influence the overall level of antibiotic resistance of naïve cells in mixed cultures. To test this hypothesis we chose to focus on the K56-2Δ*rpoE* mutant, as this bacterium has a PmB resistance profile that is similar to the parental strain but sufficiently less resistant to reach higher levels of growth inhibition at testable concentrations of PmB (Figure 1A). Based on the K56-2Δ*rpoE* PAP, we selected the subpopulation of K56-2Δ*rpoE* exposed to 500 µg/ml (Figure 1A, Δ*rpoE*/500), which arose at a frequency of 2.48×10^−4^ and demonstrated uniform high-level resistance when re-exposed to PmB (Figure 1A). Δ*rpoE*/500 cells passaged for up to five days in the absence of PmB displayed PAP identical to that of cells grown overnight in the presence of 500 µg/ml PmB, indicating that the high-level resistance of Δ*rpoE*/500 was stable without selective pressure, likely as a result of one or more mutations that confer increased PmB resistance. No differences were found between Δ*rpoE*/500 and naïve Δ*rpoE* cells in LPS electrophoretic profiles (Figure S1A). Furthermore, the increased resistance of Δ*rpoE*/500 was not due to an increase in the Ara4N LPS modification, since the differential expression of *arnT* and *arnB* genes, representing the 2 transcriptional units of the *arn* cluster [11], was 1.08±0.09) and −1.73±0.04) respectively, as determined by qRT-PCR. This was expected since we have previously shown that the *arn* cluster in *B. cenocepacia* is not regulated by PmB challenge [11]. Δ*rpoE*/500 cells treated with PmB also displayed reduced metabolic activity at 24 h relative to naïve Δ*rpoE* and K56-2 with or without exposure to PmB, as revealed in the resazurin metabolic assay (Figure S1B), suggesting that increased PmB resistance in the Δ*rpoE*/500 subpopulation is associated with reduced metabolic fitness.

**Figure 2:**
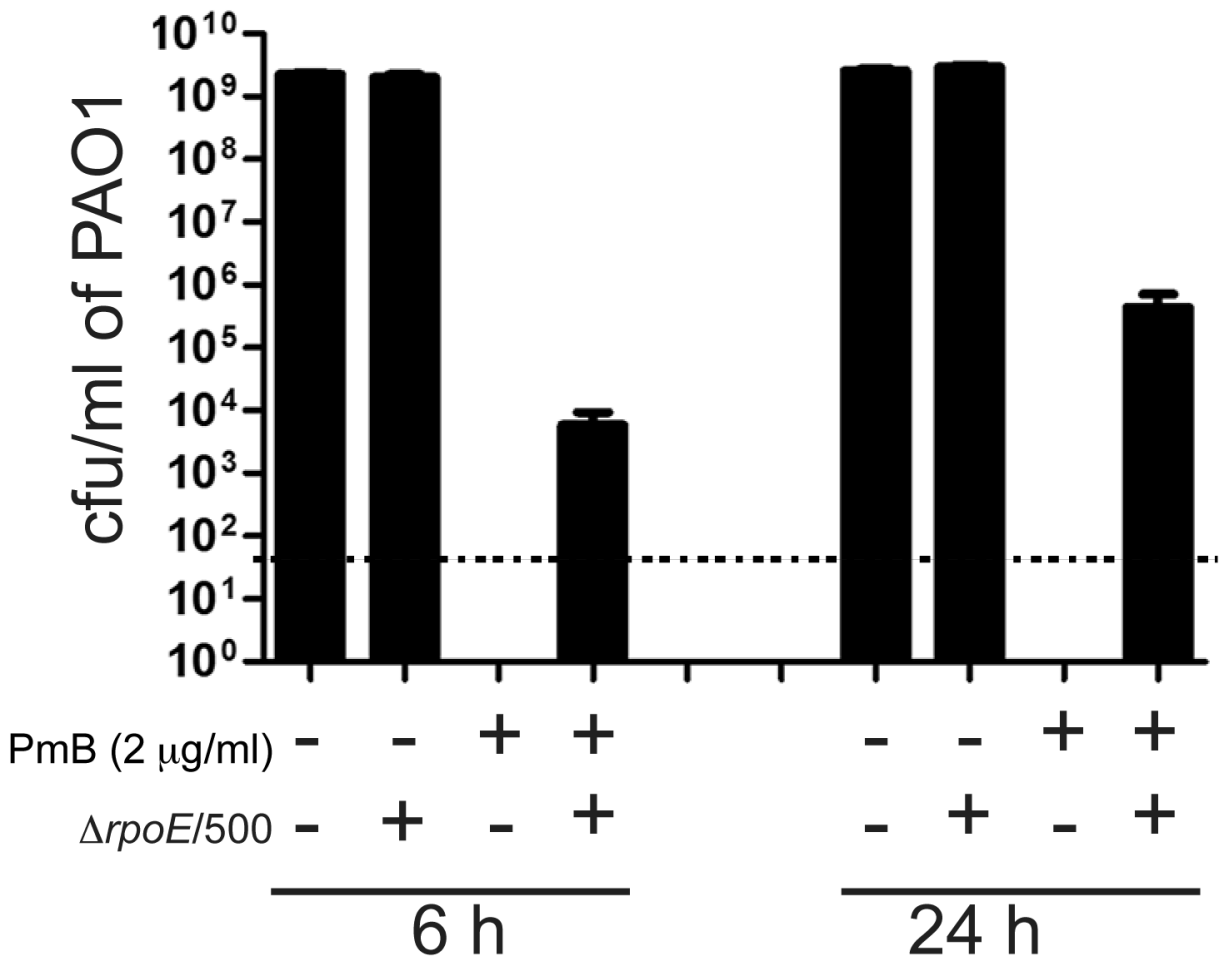
Protective effects of *B. cenocepacia* Δ*rpoE*/500 on *P. aeruginosa* PAO1, exposed to PmB, in co-culture. The dotted line represents the limit of detection (50 cfu/ml). Three independent experiments each done in duplicate.

Since Δ*rpoE*/500 represents ∼1% of the Δ*rpoE* population in the turbidimetric PAP experiments (not shown), Δ*rpoE*/500 was co-cultured in a 1∶100 ratio with *P. aeruginosa* in the presence of 2 µg/ml of PmB. This concentration of PmB was based on the current clinical guidelines for *P. aeruginosa* therapeutic breakpoints of the closely related antibiotic polymyxin E (colistin), which is set at 2 µg/ml [12] and is equivalent to the MIC of PmB against *P. aeruginosa*. Co-culture under these conditions resulted in more than a 3- to 5-log survival of *P. aeruginosa* at 6 and 24 h, respectively, compared to *P. aeruginosa* grown alone (Figure 2). There was no effect of *P. aeruginosa* on the growth of *B. cenocepacia* cells in co-culture (Figure S2). Protection by Δ*rpoE*/500 did not depend on secreted extracellular proteases since no differences were found between Δ*rpoE*/500 and naïve Δ*rpoE* cells in the amount of these proteases (Figure S1C). Similarly, protection did not depend on quorum sensing molecules, as mutants defective in the various quorum systems of *B. cenocepacia* also showed heteroresistance to PmB and could protect *P. aeruginosa* from PmB (Figure S3A and B). Also, it could not be due to production of indole [3], [4] since *B. cenocepacia* and *Burkholderia* in general are indole negative [13].

Furthermore, the filtered supernatant of an overnight culture of Δ*rpoE*/500 in PmB communicated higher-level resistance in a volatile-mediated manner to physically separated K56-2Δ*arnBC* and several *E. coli* strains. The MIC of the PmB-sensitive strains doubled due to volatiles emitted from the supernatant of Δ*rpoE*/500 (Table S1), with the exception of *E. coli* GT115, which only showed slight enhancement in the growth in the presence of PmB (not shown). These results were consistent and reproducible. The protective effect of Δ*rpoE*/500 was therefore not limited to the same species and could be communicated by one or more volatile compounds in the bacterial supernatant.

### The More Resistant Subpopulation Releases Higher Amounts of a Subset of Proteins upon Exposure to PmB

**Figure 3:**
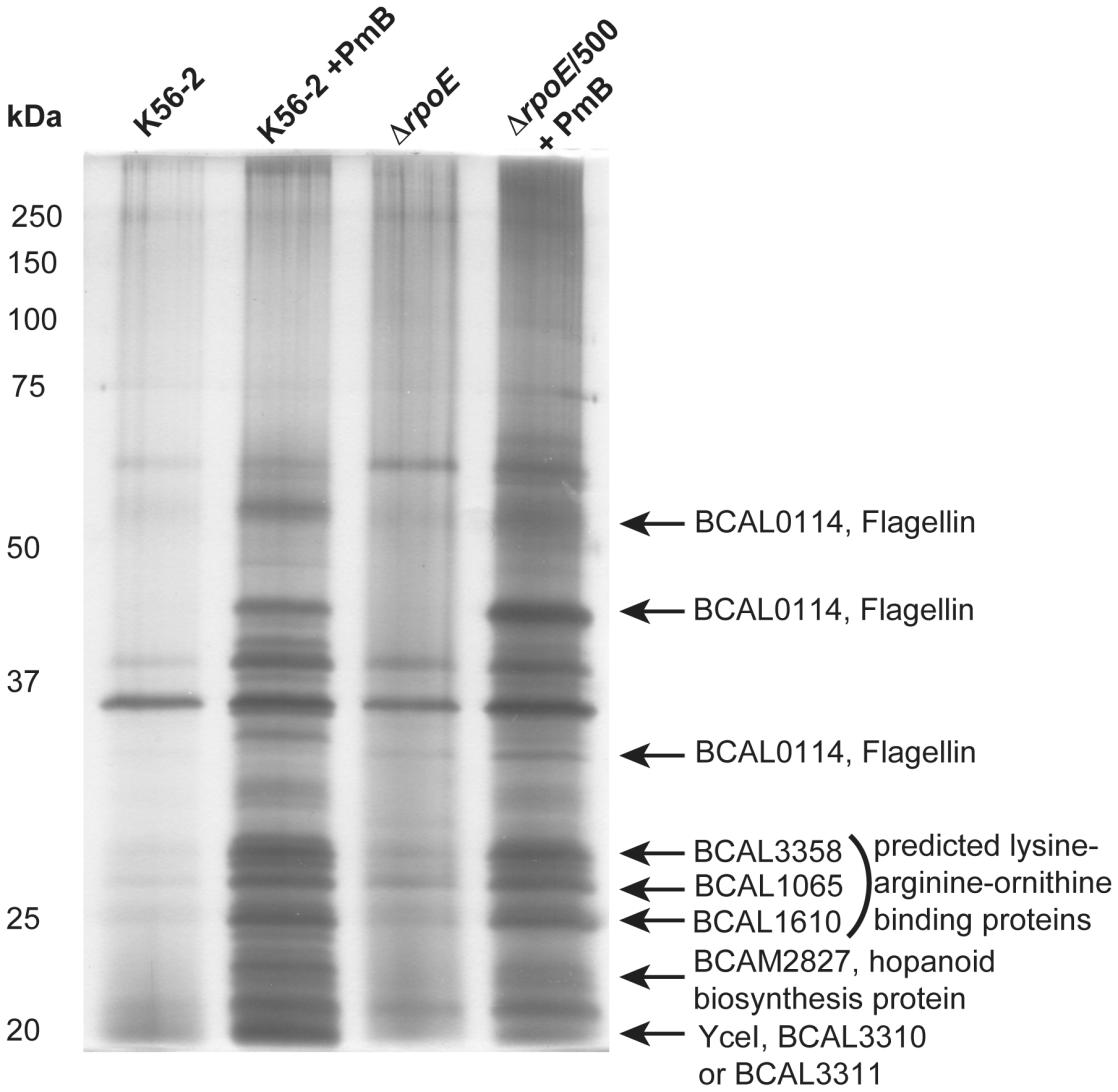
Proteins released into the supernatant of *B. cenocepacia* **K56-2 and Δ** *rpoE*/500 treated with 500 µg/ml of PmB compared to those released from untreated K56-2 and naïve Δ*rpoE*. Proteins were run on 14% SDS-PAGE and detected by silver staining and those that were differentially expressed were further identified by LC-MS.

To gain clues on the secreted molecules mediating the protective effects of *B. cenocepacia* from PmB, we compared the profile of proteins released into the supernatant of PmB-treated Δ*rpoE*/500 with naïve K56-2Δ*rpoE* and parental K56-2 treated or untreated with PmB. The Δ*rpoE*/500 subpopulation and K56-2 treated with PmB showed a similar pattern of overexpression of several polypeptide bands (Figure 3), which were identified by mass spectrometry. One of these bands corresponded to BCAM2827, which is a predicted periplasmic component of an ABC transporter involved in the biosynthesis of hopanoids. Hopanoids, bacterial substitutes of eukaryotic cholesterol that stabilize membranes and regulate membrane fluidity and permeability, have been recently shown to be required for PmB resistance in *B. cenocepacia* [14]. Another protein band was identified as YceI, a conserved protein of unknown function proposed to bind amphiphilic molecules and sequester toxic fatty acids or amides [15]. Two highly related YceI homologues, BCAL3310 and BCAL3311, are present in K56-2. Other polypeptides were identified as flagellin, in agreement with the reported effects of PmB on the flagellar assembly apparatus at the transcriptional level [16], and with the reduced motility in parental K56-2 and Δ*rpoE*/500 upon exposure to PmB (not shown).

Three other polypeptide bands were identified as lysine-arginine-ornithine-binding periplasmic proteins, which are involved in the import of these amino acids. We hypothesized that increased import of lysine, arginine and ornithine could be utilized in the modification of the membranes through the formation of lysylphosphatidylglycerol and ornithine-lipid derivatives, since modification of bacterial membranes with cationic molecules reducing their overall negative charge is one of the common mechanisms of increasing resistance to antimicrobial peptides [17]. However, deletion of *BCAM1679* encoding a putative lysylphosphatidylglycerol synthetase and *olsB* (*BCAL1281*), previously shown to render the cells incapable of synthesizing the ornithine-lipid under low phosphate conditions [18], did not affect the resistance to PmB when tested in LB medium or in low-phosphate containing medium in K56-2 background (not shown).

### A Role for Putrescine in PmB Resistance

**Figure 4:**
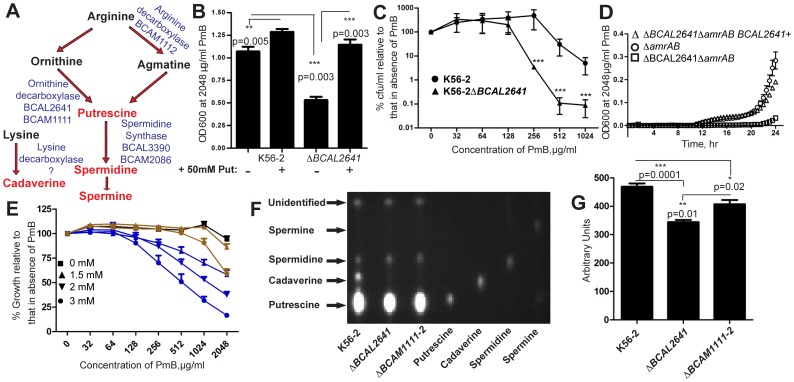
Contribution of the polyamine putrescine in the response to PmB. (A) Polyamines biosynthetic pathway. (B) Exogenous putrescine increases the resistance of the parental K56-2 to PmB and restores the resistance to PmB to the parental level in Δ*BCAL2641*; n = 6 (C) The deletion of *BCAL2641* leads to reduced resistance to PmB relative to the parental K56-2. (D) Single-copy complementation of Δ*BCAL2641*. (E) The polyamine synthesis inhibitor dicyclohexylamine (blue) reduces the resistance of *B. cenocepacia* K56-2 to PmB, with little to no effect of 3-(methylthio)propylamine (red), shown in a turbidimetric PAP at 24 h; n = 5. (F) TLC analysis of polyamines released in the supernatants of 20 h old M9 cultures compared to standards. (G) Relative amounts of putrescine released from the wild-type and mutants, n = 4.

The increased import of lysine, arginine and ornithine suggested by the overexpression of their periplasmic binding proteins in Δ*rpoE*/500 exposed to PmB could be also utilized for synthesis of polyamines (Figure 4A). Therefore, we tested the involvement of polyamines as possible candidate molecules conferring increased PmB resistance. Spermidine, at concentrations ranging from submicromolar to millimolar levels, had negligible effect on resistance of *B. cenocepacia* to PmB (not shown). However, treatment of the parental K56-2 with 50 mM putrescine increased the resistance to PmB since putrescine-treated cells survived better at 2,048 µg/ml PmB compared to control cells (Figure 4B). Putrescine treatment of K56-2Δ*arnBC* also resulted in a 2-fold increase in the MIC of PmB, suggesting that putrescine plays a role in the increased resistance to PmB and its transfer among the bacterial population.

To test this notion, we deleted the genes encoding key enzymes for polyamines biosynthesis (Figure 4A). Mutants with double deletions of both genes encoding spermidine synthases (BCAL3390 and BCAM2086) showed a slight reduction in resistance to PmB and no changes in growth rate (Figure S4). However, the mutant lacking *BCAL2641*, which encodes an ornithine decarboxylase, had a marked reduction in resistance to PmB and no growth rate defects (Figure 4C). PmB resistance was restored to parental level by single-copy complementation of *BCAL2641* (Figure 4D). To further confirm these findings we used two polyamines synthesis inhibitors, dicyclohexylamine and 3-(Methylthio)propylamine (Figure 4E and Figure S5). Dicyclohexylamine, originally reported as a spermidine synthase inhibitor but also capable of inhibiting the ornithine decarboxylase enzyme (Figure S6), reduced resistance to PmB in K56-2 and Δ*rpoE*/500 cells in a concentration dependent manner (Figure 4E and Figure S5, respectively). However, 3-(Methylthio)propylamine, which is more specific for spermidine synthase and lacks any detectable inhibitory effect on the ornithine decarboxylase reaction (Figure S6) had no effect on resistance of Δ*rpoE*/500 (Figure S5) and only caused a lower reduction of resistance of K56-2 to PmB (Figure 4E). Exogenous putrescine increased the resistance of K56-2 Δ*BCAL2641* to PmB in a concentration-dependent manner; full restoration of the level of resistance of the parental strain was achieved at 50 mM putrescine (Figure 4B). A comparison of the level of transcription of *BCAL2641* in both naïve Δ*rpoE* and Δ*rpoE*/500 bacteria treated with PmB, demonstrated that the expression of this gene is upregulated by 2.9±0.9 in Δ*rpoE*/500 in response to PmB. Moreover, higher levels of putrescine released in the supernatant of Δ*rpoE*/500 treated with PmB were observed relative to Δ*rpoE* naïve population (Figure S7).

**Figure 5:**
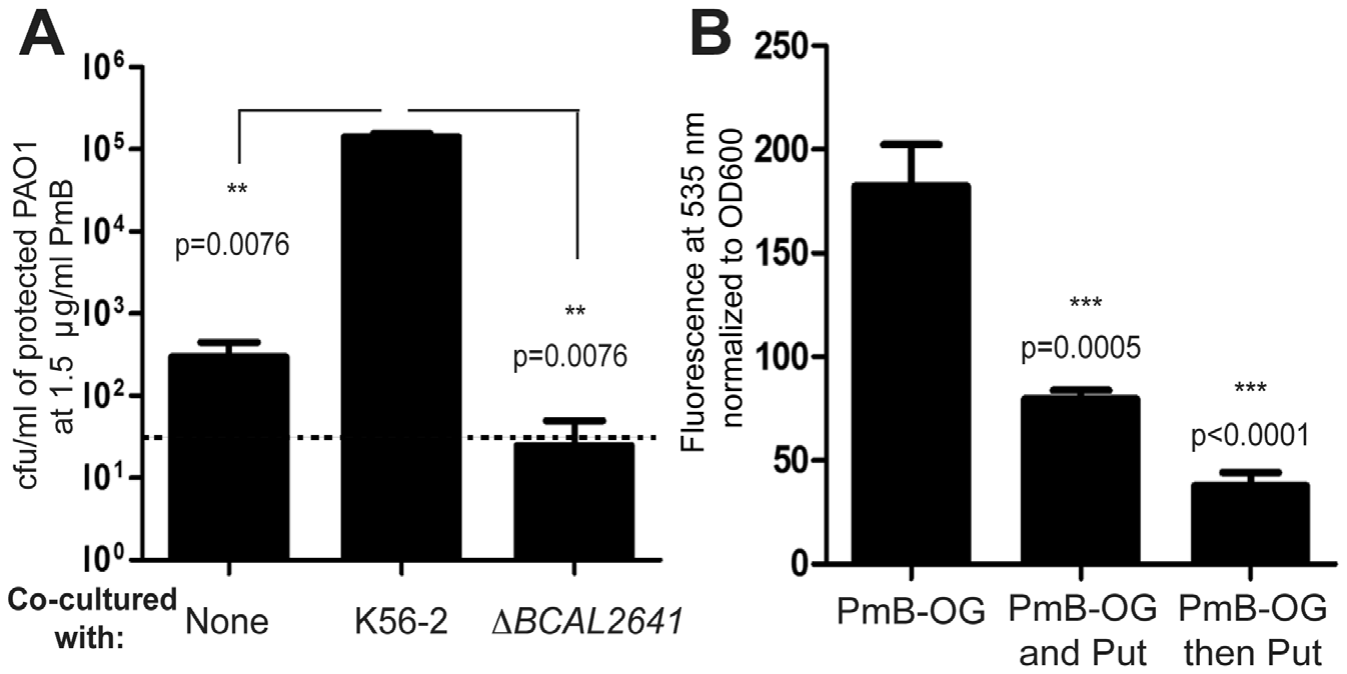
Role of putrescine in the protective effects of *B. cenocepacia* against PmB. (A) Involvement of putrescine in the protective effects of *B. cenocepacia* on *P. aeruginosa* PAO1 at 24 h. The dotted line represents the limit of detection (50 cfu/ml). Three independent experiments each done in duplicate. (B) Putrescine protects the bacterial surface from binding to PmB; 50 mM of putrescine reduced binding of PmB-Oregon green 514 conjugate (25 µg/ml) when both added together, whereas it could replace already bound PmB; n = 6.

Putrescine is the most abundant polyamine secreted from *B. cenocepacia*, while much less amounts of spermidine and cadaverine are secreted from K56-2 (Figure 4F). The release of putrescine was significantly reduced in the Δ*BCAL2641* compared to the wild type K56-2 (Figure 4G). However, *B. cenocepacia* possesses another predicted ornithine decarboxylase, BCAM1111 and a putative arginine decarboxylase, BCAM1112. Deletion of genes encoding both enzymes did not have an effect on resistance to PmB in K56-2 (not shown), and only a small effect in the release of putrescine (Figure 4G). In contrast, cadaverine was not detected in the supernatant of Δ*BCAL2641* and Δ*BCAM1111*Δ*BCAM1112* precluding the involvement of cadaverine in increased PmB resistance (Figure 4F). By qRT-PCR, *BCAM1111* and *BCAM1112* were 2000-fold less transcribed relative to *BCAL2641* in naïve Δ*rpoE* and Δ*rpoE*/500 bacteria; they were also not differentially transcribed in the more resistant subpopulation (Δ*rpoE*/500) relative to the naïve population, suggesting that their gene products are not preferentially used in polyamines biosynthesis. In agreement, the ornithine decarboxylase (ODC) activity of Δ*BCAL2641* was much more reduced relative to Δ*BCAM1111*Δ*BCAM1112* (Figure S8). The pattern of ODC activity corresponded to the levels of secretion of putrescine in the different mutants relative to the wild type (Figure 4G). Together, this shows that BCAL2641 is the primary contributor of putrescine in *B. cenocepacia* explaining the phenotype observed upon its deletion. K56-2 Δ*BCAL2641* also lost the protective effects from PmB in co-culture with *P. aeruginosa* PAO1 (Figure 5A). These results implicated putrescine as a critical polyamine conferring protection from PmB and communicating resistance to neighbouring bacterial cells.

The initial binding of antimicrobial peptides to the bacterial surfaces is crucial for their subsequent antibacterial effects [17]. Putrescine competed with PmB for binding to the surface of *B. cenocepacia* K56-2, where treatment of cells with both putrescine and the fluorescent PmB- Oregon green 514 conjugate showed reduced binding of the fluorescent PmB derivative relative to control cells (Figure 5B). Putrescine also replaced already bound fluorescent PmB conjugate (Figure 5B). This agrees with previous findings showing that polyamines provide protection of the outer membrane of *P. aeruginosa* from PmB damage [19]. However, this does not preclude other mechanisms mediated by putrescine to protect against the effects of PmB. For example, polyamines can reduce oxidative stress in *E. coli* exposed to bactericidal antibiotics [20] and protect from membrane lipid peroxidation in *P. aeruginosa* [19]. These additional mechanisms of protection by polyamines are consistent with the notion that bactericidal antibiotics at sublethal concentrations stimulate the production of hydroxyl radicals, which in turn may induce mutations leading to various levels of antibiotic resistance [21].

### The Role of YceI Protein

**Figure 6:**
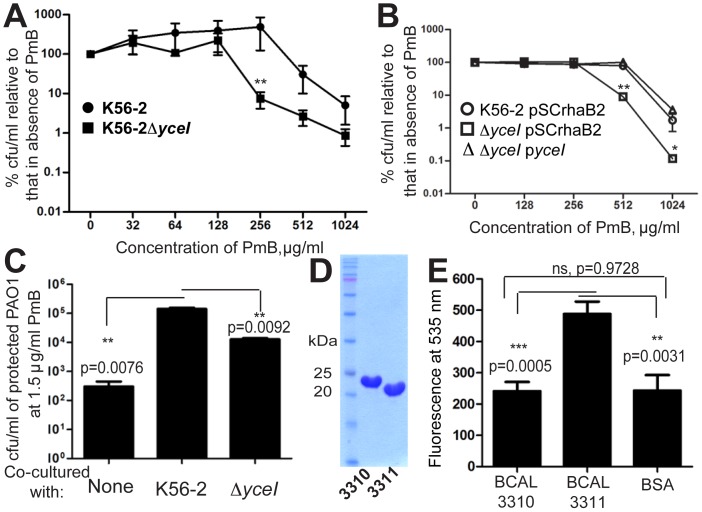
Contribution of YceI in the response to PmB and its role in protection against PmB. (A) The deletion of *BCAL3310* and *BCAL3311* (Δ*yceI*) leads to reduced resistance to PmB relative to the parental K56-2. (B) PAP by agar dilution showing complementation of the reduced resistance in Δ*yceI* mutant by *yceI* under the control of the rhamnose promoter on pSCrhaB2 to the parental level at 0.4% rhamnose. (C) Involvement of YceI in the protective effects of *B. cenocepacia* on *P. aeruginosa* PAO1 at 24 h. Three independent experiments each done in duplicate. (D) Purified YceI homologues, BCAL3310 and BCAL3311. (E) Binding of BCAL3310 and BCAL3311 to PmB-Oregon green 514 conjugate. BSA was used as a control for binding. n = 6.

We also tested the involvement of YceI in heteroresistance. Mutants with a double deletion of *BCAL3310* and *BCAL3311* had increased sensitivity to PmB, but no differences in growth rate relative to K56-2 (Figure 6A). Complementing the double deletion mutant Δ*BCAL3310*Δ*BCAL3311* (K56-2Δ*yceI*) with both genes restored resistance to PmB to the parental level (Figure 6B). Moreover, YceI contributed to the protective effects of *B. cenocepacia* towards *P. aeruginosa* PAO1 cells exposed to 1.5 µg/ml PmB (Figure 6C). The level of transcription of BCAL3310, determined by qRT-PCR in both naïve Δ*rpoE* and the more resistant subpopulation Δ*rpoE*/500 treated with PmB, indicated that this gene was upregulated by 2.5±0.6 in the more resistant subpopulation in response to PmB. Together, these experiments reveal that the YceI homologues contribute to the increased resistance to PmB in Δ*rpoE*/500 and the protective effects on other cells against PmB. Purified YceI BCAL3310 and BCAL3311 (Figure 6D), were both capable of binding PmB-Oregon green 514 conjugate, although BCAL3311 being more potent than BCAL3310 (Figure 6E). This supports their role in sequestering PmB thus protecting other cells from the toxic effects of the antibiotic.

### *B. cenocepacia* is Heteroresistant to other Bactericidal Antibiotics

**Figure 7:**
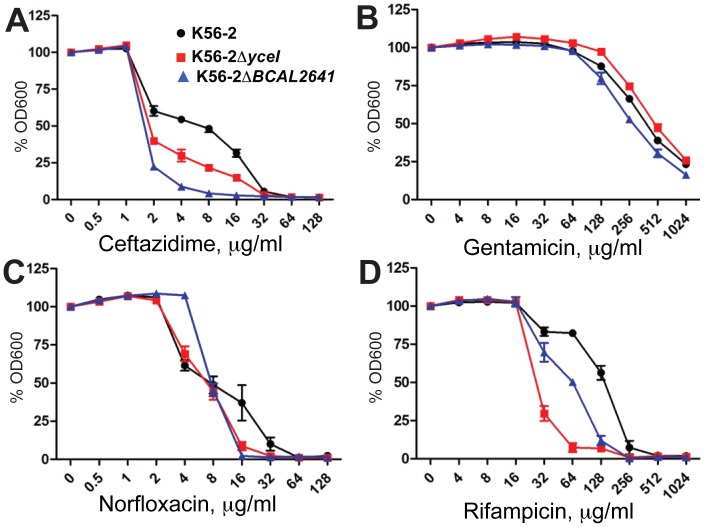
****** ****

**Figure 8:**
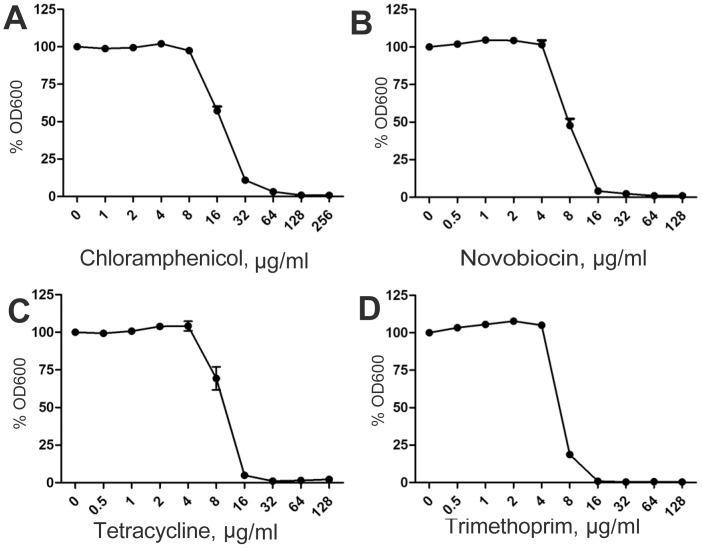
Homogenous response of *B. cenocepacia* K56-2 to bacteriostatic antibiotics. n = 6.

We determined whether heteroresistance in K56-2 is exclusive to PmB. Turbidimetric PAP using various antibiotics indicated that K56-2 is heteroresistant to gentamicin (protein synthesis inhibitor), norfloxacin (DNA replication inhibitor), rifampicin (mRNA transcription inhibitor) and ceftazidime (cell wall peptidoglycan synthesis inhibitor), all of which belong to different classes of bactericidal antibiotics (Figure 7). In contrast, the response of K56-2 was homogeneous to tetracycline, chloramphenicol, novobiocin, trimethoprim, which are all bacteriostatic antibiotics (Figure 8). Polyamines play a role in the heterogeneity of response to the bactericidal antibiotics. Δ*BCAL2641* displayed a more homogeneous response to the different bactericidal antibiotics, except for gentamicin (Figure 7). Similarly, YceI was involved in the heterogeneous response to the amphiphilic bactericidal antibiotics rifampicin and norfloxacin; however, the Δ*yceI* mutant only showed minor reduction in the percentage of the more resistant fractions of the population in response to ceftazidime (Figure 7).

### Conclusions

We show that (i) *B. cenocepacia* is heteroresistant to PmB and different classes of bactericidal antibiotics; (ii) a more resistant subpopulation of *B. cenocepacia* communicates high-level resistance to less resistant cells; (iii) the protection extends to other bacterial species and is chemically mediated by putrescine, a polyamine, and the secretion of YceI. Since putrescine is volatile [22], resistance can also be communicated to physically separated bacteria in a volatile-mediated manner. Natural polyamines, discovered more than 300 years ago, occur in almost all living organisms; they are involved in growth, development, and other important functions related to modulation of defence responses to diverse environmental stresses and modulation of immune responses in plants and humans respectively [23], [24]. Polyamines are significantly increased at inflammatory sites of infection or injury [25], [26]; they are also produced by a wide range of bacteria, playing roles in growth and other functions including incorporation into the cell wall, biosynthesis of siderophores, acid resistance, scavenging free radical ion, signaling cellular differentiation and biofilm formation [27]. The two most common bacterial polyamines are putrescine and spermidine [27]. We show here that the most abundant polyamine in *B. cenocepacia* is putrescine, while spermidine and cadaverine are produced in much lower amounts. Polyamines were previously shown to increase the resistance of *P. aeruginosa* to antimicrobial peptides [19], [28], [29]. Heteroresistance of *B. cenocepacia* K56-2 was common to bactericidal antibiotics regardless of their site of action. We speculate that bacterial cells may be exposed to greater stress in case of bactericidal agents, relative to bacteriostatic antibiotics, which might result in variation across the bacterial population in their capabilities to withstand and respond to such hostile insult. The involvement of polyamines in heteroresistance to the different classes of bactericidal antibiotics and of YceI in the response to amphiphilic bactericidal antimicrobials leads us to propose that these mediators serve as "danger" infochemicals. These chemical signals may be employed in the non-genetic communication of resistance among members of heteroresistant bacterial populations against the different bactericidal antibiotics. The action of YceI on amphiphilic antibiotics fits with its proposed mechanism sequestering toxic amphiphiles with acyl fatty chains, such as PmB, as we have shown in this study. However, this does not preclude other mechanisms in the response of *B. cenocepacia* to bactericidal antibiotics, especially the aminoglycoside gentamicin, which still requires further investigation.

The proposed danger infochemicals can serve as a general mechanism of protection of other bacterial species in a polymicrobial infection such as that found in patients with cystic fibrosis. YceI would reduce available amphiphilic antibiotics from the medium thus protecting any organism; whereas putrescine could interact with most of the bacterial species, since polyamines are produced by most bacteria, with rare exceptions such as *Staphylococcus aureus* strains, which do not tolerate polyamines as they lack polyamines detoxifying enzymes [30].

In conclusion, we show that antibiotic heteroresistance leads to a cooperative behaviour such that the more antibiotic-resistant members of the population protect the less resistant ones as well as less resistant members of other species. A similar observation has been made previously with indole production by *E. coli* strains [3]. However, indole production in the more resistant cells was at the exact same level as in naive cells with no antibiotic treatment and unlike putrescine, indole was neither induced by antibiotics nor over-secreted by the more resistant cells. We believe our findings are relevant in the clinical setting, particularly for intrinsically resistant opportunistic Gram-negative bacteria. Attempts to modulate these interactions using polyamine synthesis inhibitors may contribute to disrupting heteroresistance so the bacterial population will have a more uniform response to the antibiotic, reducing the window of therapeutic failure.

## Materials and Methods

### Strains and Reagents

Table S2 lists bacteria and plasmids used in this work. Bacteria grew in LB at 37°C. Antibiotics (Sigma, St Louis, MO, USA) were diluted in water except for PmB, which was diluted in 0.2% bovine serum albumin/0.01% glacial acetic acid buffer. For growth analyses, overnight cultures were diluted to an optical density at 600 nm (OD_600_) of 0.0008 and incubated at 37°C with medium continuous shaking in a Bioscreen C automated growth curve analyzer (MTX Lab Systems, Vienna, VA, USA). Medium 121 containing 83 µM phosphate was used to test low phosphate conditions [31]. Extracellular protease production was determined on dialyzed Brain-Heart infusion milk agar plates [32]. Lipopolysaccharide was extracted and visualized by silver staining [33]. Etest strips (AB bioMérieux, Solna, Sweden) were applied to agar plates inoculated with test bacteria by swabbing overnight cultures diluted to OD_600_ of 0.04; plates were then incubated at 37°C for 24 h. Unmarked non-polar deletions were performed as described previously [34]. Unmarked chromosomal single copy complementation of *BCAL2641* was performed using pMH447 [10]. Complementation of *yceI* (*BCAL3310* and *BCAL3311*) was performed using pSCrhaB2 [35].

### Population Analysis Profiling (PAP)

This involved treating bacterial cultures with doubling increments of antibiotic concentrations and determining growth at each concentration by turbidimetry in LB broth (PAP by broth dilution) or by cfu counting on agar plates (PAP by agar dilution). Heteroresistance was considered when the antibiotic concentration exhibiting the highest inhibitory effects was 8-fold or more higher than the highest non-inhibitory concentration.

### Co-culture

Co-culture was performed by mixing overnight cultures of *P. aeruginosa* PAO1 and *B. cenocepacia* (treated with 500 µg/ml PmB) diluted to OD_600_ of 0.004 at ratio 100∶1 in LB broth with or without PmB. Controls with the pure cultures at the same inoculum size were included in the experiment. The mixtures were incubated at 37°C at 200 rpm and cfu of each species was determined by using differential antibiotic selection on LB agar plates at 6 and 24 h. *B. cenocepacia* was selected with PmB (50 µg/ml) and PAO1 was selected with trimethoprim (100 µg/ml). The total count was determined on LB agar plates.

### Volatile-mediated Protection

Overnight culture of *B. cenocepacia* was diluted 1 in 200 in LB containing 500 µg/ml PmB and incubated at 37°C for 17 h at 200 rpm. The supernatant was collected at 4°C, filtered using 0.2 µm nylon membrane filters, and 10 ml aliquots were placed at one side of the septum in septate Petri dishes. MIC by agar dilution was performed on test bacteria (*B. cenocepacia* K56-2Δ*arnBC, E. coli* DH5α, HB101, and GT115) at the other side of the septum by spotting (10 µl) of their overnight cultures diluted to OD_600_ of 0.004 on LB agar containing PmB at doubling increments. The plates were then incubated at 37°C for 24 h.

### RNA Extraction

Δ*rpoE*/500 and Δ*rpoE* cells were grown overnight and then diluted to OD_600_ of 0.05 in 50 ml of LB with 500 µg/ml PmB or vehicle control respectively. Cells were grown at 37°C for 30 min at 200 rpm then collected by centrifugation at 39,000×*g* for 30 min at 4°C. RNA was prepared from approximately 5×10^8^ cfu using the RiboPure-Bacteria kit (Ambion, Inc., Austin, TX, USA) and treated with DNAse I (Ambion), followed by treatment with DNAse I (Qiagen Inc., Mississauga, ON, Canada) following the manufacturer’s protocol. Integrity of the RNA was assessed by agarose gel electrophoresis and by measuring the ratio of absorbance at 260 nm to 280 nm (values obtained between 2.0 and 2.2).

### qRT-PCR

RNA was converted to cDNA and real-time PCR was performed as previously described [36]. Fold changes in gene expression were calculated using the Pfaffl Method [37] relative to *BCAS0175*, an internal control used for microarray and real-time PCR analysis [38]. Data were calculated from 3 independent experiments each done in triplicate.

### Ornithine Decarboxylase (ODC) Assay

Overnight cultures in LB broth were diluted to OD_600_ of 0.004 in the rapid ornithine broth medium described by Fay and Barry [39] with or without PmB or the polyamine synthesis inhibitors adjusted at pH 5.5. Aliquots (300 µl each) were transferred to 100-well Bioscreen C plates and overlaid with 100 µl of mineral oil. The plates were incubated in the Bioscreen C automated growth curve analyzer at 37°C without shaking and the color was monitored at 420 nm.

### Thin-layer Chromatography Analyses of Polyamines

Polyamine analysis was performed as previously described [40]. Overnight cultures (∼20 h) in M9 medium with or without PmB were used. Supernatants, collected by centrifugation at 16,100×*g* for 5 min, corresponding to cultures of OD_600_ of 0.1 were used. HClO_4_ (4 N) was added to supernatants to reach a final normality of 0.4 N and incubated at 37°C for 1 h with shaking. HClO_4_ extracts were centrifuged at 16,100×*g* for 5 min. Fifty microlitres of the supernatants were mixed with 50 µl of 2 M Na_2_CO_3_ and 100 µl of 2.7-mg/ml dansyl chloride (Sigma, St Louis, MO, USA) solution in acetone and incubated in the dark at 37°C for 2 h with shaking. Standard solutions of putrescine, cadaverine, spermidine and spermine (0.2 mM each) were treated similarly. The mixtures were evaporated to dryness under Nitrogen gas and extracted with 200 µl benzene at 4°C for ∼18 h with shaking. Fifty microlitres of the benzene extracts of each of the samples and 5 µl of each of the standards were applied onto TLC silica gel plates (20×20 cm, Merck, Darmstadt, Germany) and sequentially separated in two systems: I) benzene–triethylamine (20∶2 v/v); II) benzene–methanol (10∶0.45 v/v). The dried plates were photographed in ultraviolet light, which excites the green-blue fluorescence of dansyl polyamine spots. The size and intensity of these spots were proportional to the polyamine concentration, which was quantified using Image J 1.46r software.

### Competition between Putrescine and Fluorescent PmB on Surface Binding

Overnight culture of *B. cenocepacia* K56-2 was centrifuged at 16,100×*g* for 1 min, and cells were washed with PBS (3x) followed by dilution to OD_600_ of 1 in PBS. Polymyxin B Oregon Green 514 conjugate, PmB-OG (Invitrogen) was added to 100 µl diluted cells at final concentration of 25 µg/ml and incubated at 37°C for 10 min. Then, cells were washed with PBS (3x), resuspended in 100 µl of PBS, and placed into 96-well white plates. Fluorescence was measured at λ_ex_ of 480 nm and λ_em_ of 535 nm. Data was reported as a ratio of Fluorescence to OD_600_.

### Cloning, Expression, and Purification of YceI

Genes encoding the two YceI homologues (BCAL3310 and BCAL3311) were individually amplified by PCR from K56-2 genomic DNA without the sequences encoding the signal peptides. The constructs were cloned into the pET28a expression vector. The positive pET28a–BCAL3310 or 3311 clones were verified by sequencing. The two YceI homologues were overexpressed in *E. coli* (BL21 strain) using 0.05 mM isopropyl thio-β-d-galactoside, and the expression was prolonged for 3 h at 25 °C. Bacterial cells were harvested and the cell pellet was resuspended in 50 mM phosphate buffer pH 7.8 and lysis was achieved using one shot cell disrupter (Constant Systems Ltd., Northants, UK) at 27 KPSI. The resulting supernatant was isolated from the insoluble fraction by centrifugation at 16,100×*g* for 60 min at 4 °C. His-tag batch purification was performed using Ni^2+^ coated beads. The purified proteins were detected by Coomassie blue staining following 16% SDS-PAGE and quantified by Bradford assay using bovine serum albumin (BSA) as standard.

### Binding Assay of YceI to PmB

Purified BCAL3310 and BCAL3311 proteins were diluted to 10 µg/ml, treated with PmB-OG at final concentration of 1 µg/ml in a total volume of 100 µl and incubated at 37°C for 10 min with rotation. The fluorescence was measured at λ_ex_ of 480 nm and λ_em_ of 535 nm. Background fluorescence of PmB-OG with the buffer control was subtracted. BSA was used as a control for non-specific binding.

### Statistical Analyses

Unpaired student’s t-tests were conducted with GraphPad Prism 5.0.

## Supporting Information

Figure S1 **Characterization of the more resistant subpopulation Δ** ***rpoE*** **/500.** (A) LPS profiles; (B) Metabolic activity. Overnight cultures were diluted to OD_600_ of 0.02, treated with PmB or vehicle control, incubated at 37°C with continuous medium shaking for 24 h in a Bioscreen C automated growth curve analyzer. Cells were then collected, washed, resuspended in PBS, transferred to white 96-well plate, and treated with resazurin at final concentration 2.5 µg/ml. The plates were incubated in the dark at 37°C for 90 min, and the fluorescence was measured at λ_ex_ of 485 nm and λ_em_ of 600 nm; (C) Secreted protease activity.
(TIF)

Figure S2 **The growth of** ***B. cenocepacia*** **Δ** ***rpoE*** **/500 in co-culture with** ***P. aeruginosa*** **PAO1.** The growth of *B. cenocepacia* **Δ** *rpoE*/500 subpopulation was not impaired in co-culture with *P. aeruginosa* PAO1 except at 24 h in co-culture without PmB where its ratio relative to PAO1 dropped 10 fold probably due to limiting nutrients as a result of the increased biomass of both bacteria in the absence of PmB.
(TIF)

Figure S3 **Quorum sensing systems of** ***B. cenocepacia*** **are neither involved in the heterogeneity of response to PmB nor in protection to naïve populations.** (A) PAP by agar dilution of the quorum-sensing mutants. (B) Direct co-culture of subpopulations of the quorum-sensing mutants growing at 500 µg/ml with *P. aeruginosa* PAO1 in comparison with Δ*rpoE*/500 subpopulation treated with 2 µg/ml PmB for 24 h; the differences are not statistically significant.
(TIF)

Figure S4 **PmB resistance of the spermidine synthase double mutant K56-2 Δ** ***BCAL3390*** **Δ** ***BCAM2086*** **.** (A) Growth curves; (B) Effect of 2,048 µg/ml PmB.
(TIF)

Figure S5 **Effects of polyamine synthesis inhibitors on PmB resistance.** The polyamine synthesis inhibitor dicyclohexylamine (blue) reduces the resistance of *B. cenocepacia* **Δ** *rpoE*/500 subpopulation to PmB, with no effect of 3-(methylthio)propylamine (red), shown in a turbidimetric PAP at 18 h; n = 5.
(TIF)

Figure S6 **Ornithine decarboxylase (ODC) activity of** ***B. cenocepacia*** **K56-2 either untreated or treated with 1 mM of dicyclohexylamine or 3-(methylthio)propylamine at 24 h.** This concentration of the polyamine synthesis inhibitors did not affect the growth of the bacteria. n = 9.
(TIF)

Figure S7 **Release of putrescine in the Δ** ***rpoE*** **/500 culture supernantant.** Increased release of putrescine in the supernatant of Δ*rpoE*/500 subpopulation treated with 500 µg/ml PmB relative to naïve Δ*rpoE* determined at 20 h from M9 cultures by TLC analysis.
(TIF)

Figure S8 **ODC assay of the parental strain K56-2 and different PAs biosynthetic mutants at 6 h. n = 9.**
(TIF)

Table S1 **MIC by agar dilution technique to determine the volatile-mediated protective effect of the supernatant of Δ** ***rpoE*** **/500 from the effects of PmB on sensitive bacteria.**
(PDF)

Table S2 **Strains and Plasmids.**
(PDF)
