# Supplementary figures and images for: Chemical Communication of Antibiotic Resistance by a Highly Resistant Subpopulation of Bacterial Cells
Source: PLoS One. 2013 Jul 3;8(7):e68874. doi: 10.1371/journal.pone.0068874 (PMC3700957; doi:10.1371/journal.pone.0068874)

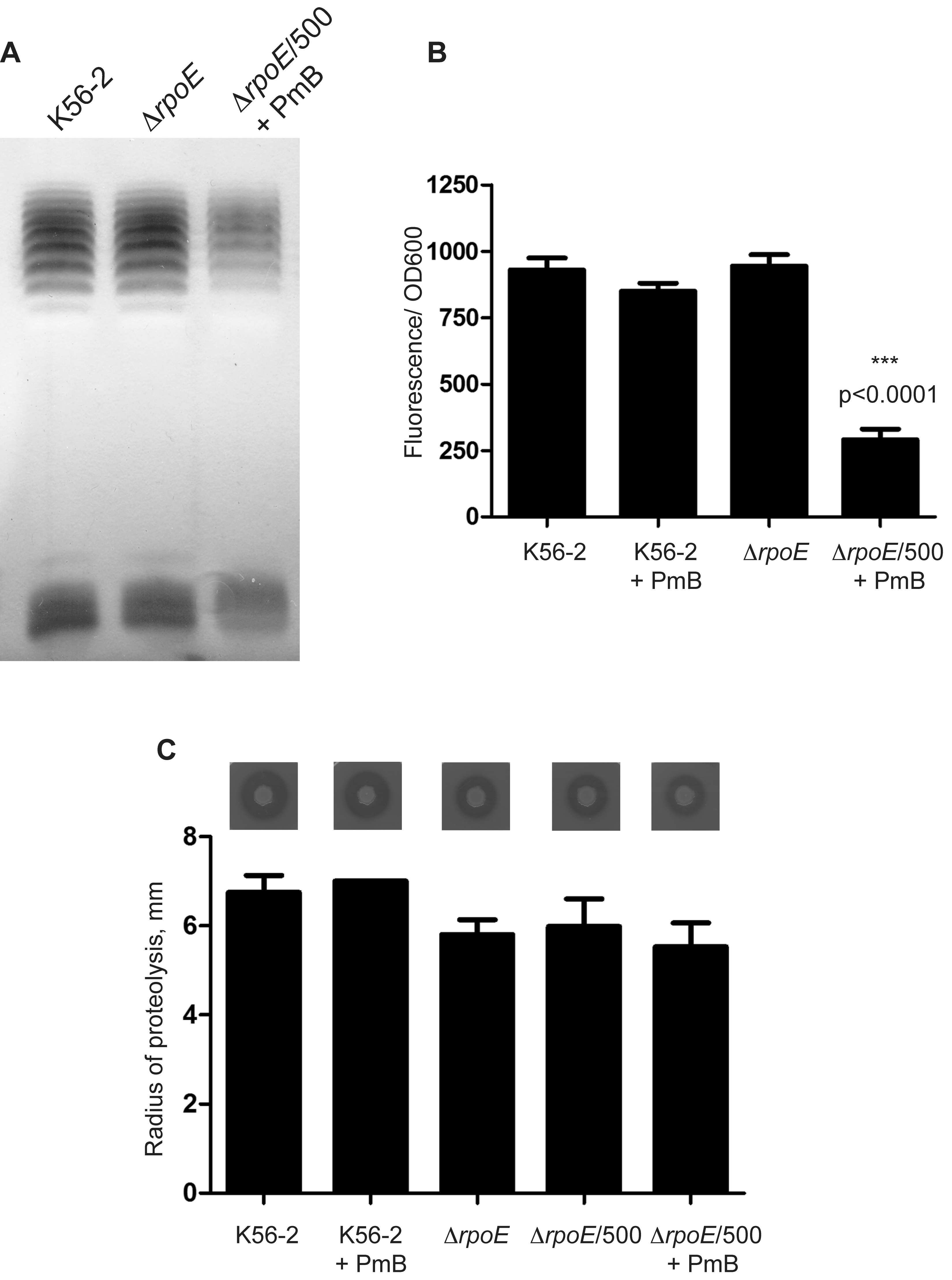

Supplement: Figure S1 — Characterization of the more resistant subpopulation Δ rpoE /500. (A) LPS profiles; (B) Metabolic activity. Overnight cultures were diluted to OD600 of 0.02, treated with PmB or vehicle control, incubated at 37°C with continuous medium shaking for 24 h in a Bioscreen C automated growth curve analyzer. Cells were then collected, washed, resuspended in PBS, transferred to white 96-well plate, and treated with resazurin at final concentration 2.5 µg/ml. The plates were incubated in the dark at 37°C for 90 min, and the fluorescence was measured at λex of 485 nm and λem of 600 nm; (C) Secreted protease activity. (TIF) [file pone.0068874.s001.tif]

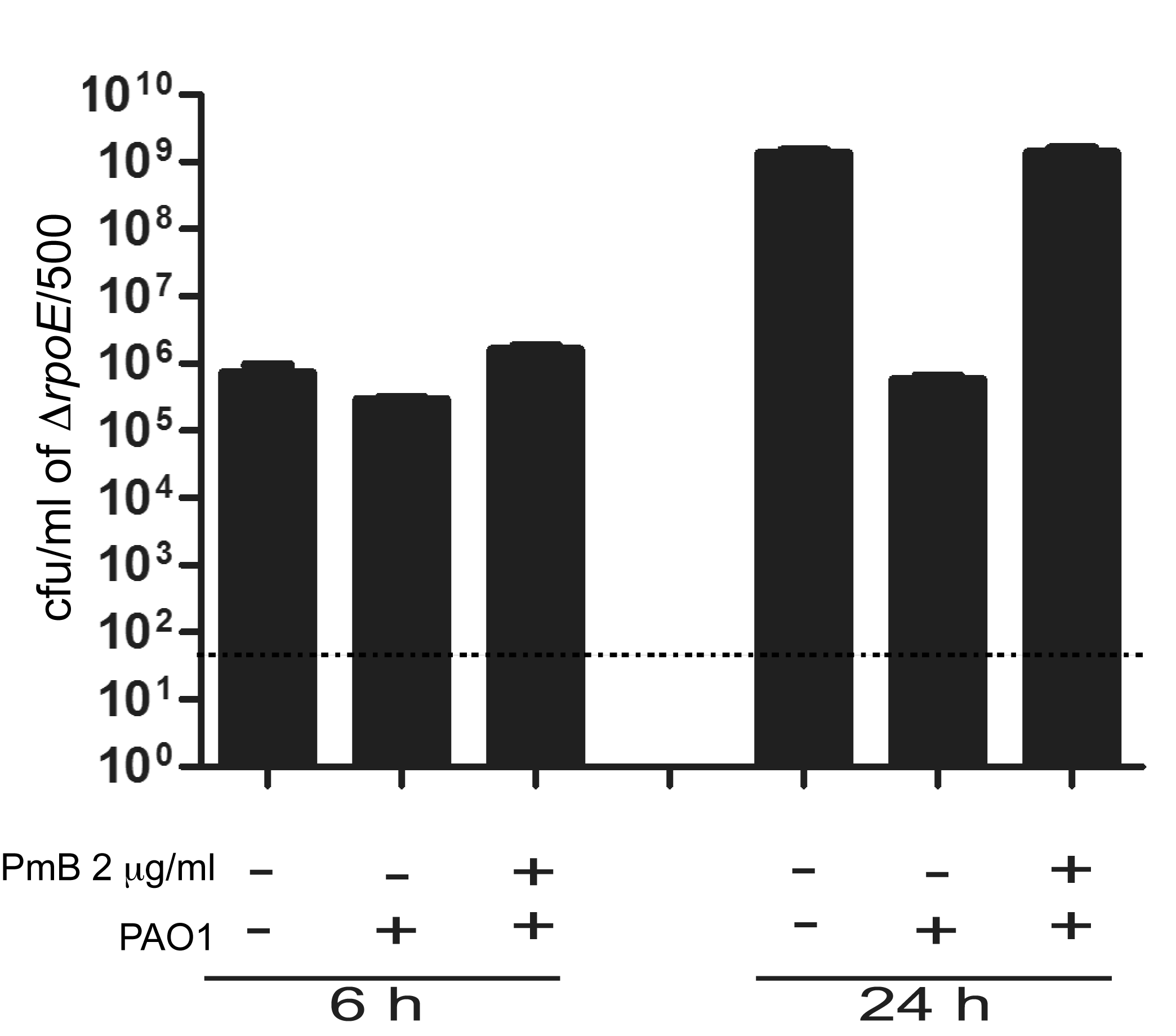

Supplement: Figure S2 — The growth of B. cenocepacia Δ rpoE /500 in co-culture with P. aeruginosa PAO1. The growth of B. cenocepacia Δ rpoE/500 subpopulation was not impaired in co-culture with P. aeruginosa PAO1 except at 24 h in co-culture without PmB where its ratio relative to PAO1 dropped 10 fold probably due to limiting nutrients as a result of the increased biomass of both bacteria in the absence of PmB. (TIF) [file pone.0068874.s002.tif]

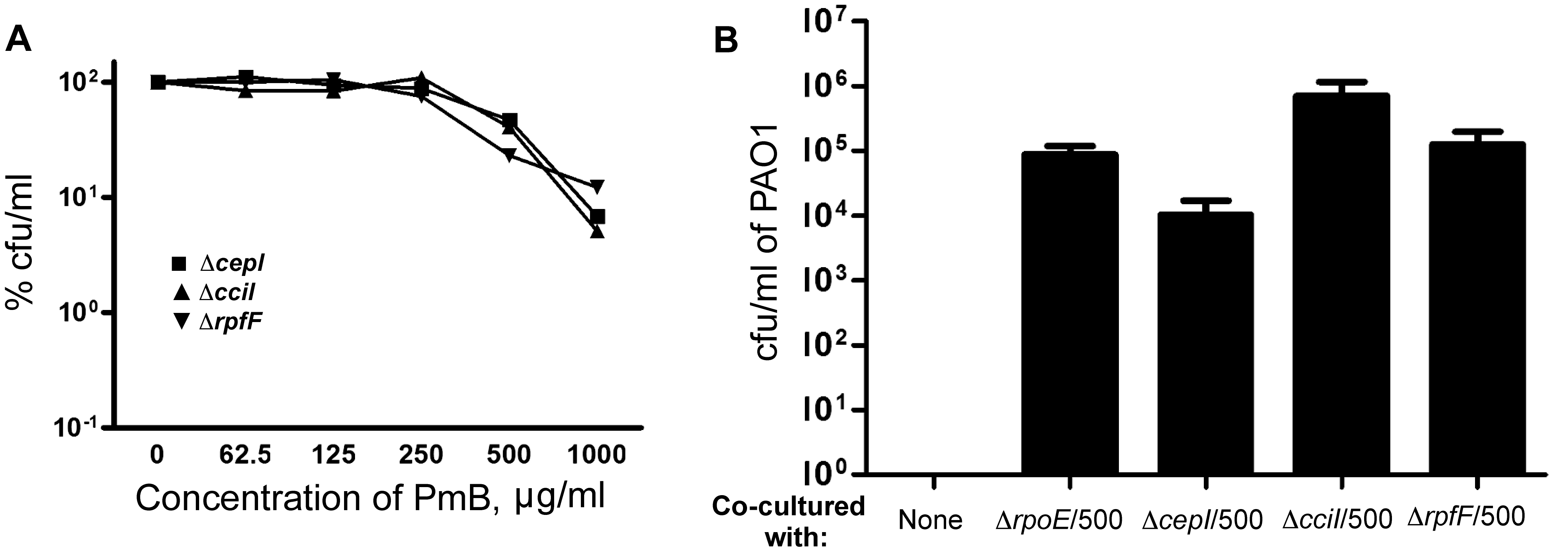

Supplement: Figure S3 — Quorum sensing systems of B. cenocepacia are neither involved in the heterogeneity of response to PmB nor in protection to naïve populations. (A) PAP by agar dilution of the quorum-sensing mutants. (B) Direct co-culture of subpopulations of the quorum-sensing mutants growing at 500 µg/ml with P. aeruginosa PAO1 in comparison with ΔrpoE/500 subpopulation treated with 2 µg/ml PmB for 24 h; the differences are not statistically significant. (TIF) [file pone.0068874.s003.tif]

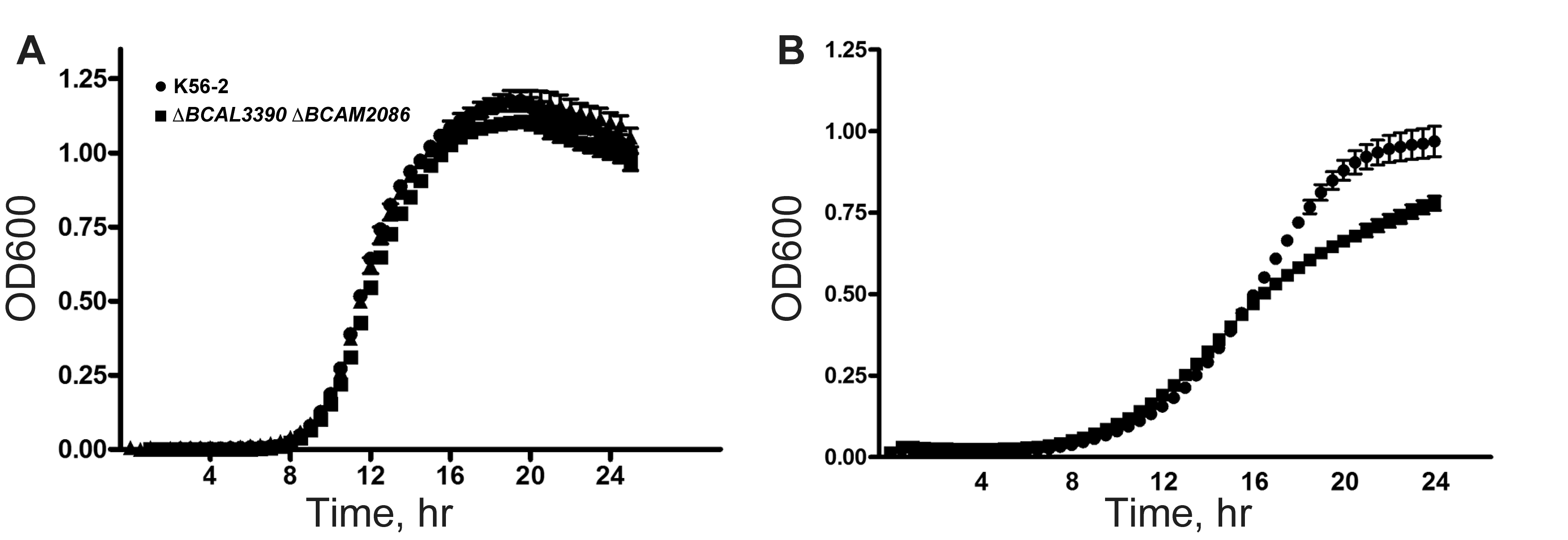

Supplement: Figure S4 — PmB resistance of the spermidine synthase double mutant K56-2 Δ BCAL3390 Δ BCAM2086 . (A) Growth curves; (B) Effect of 2,048 µg/ml PmB. (TIF) [file pone.0068874.s004.tif]

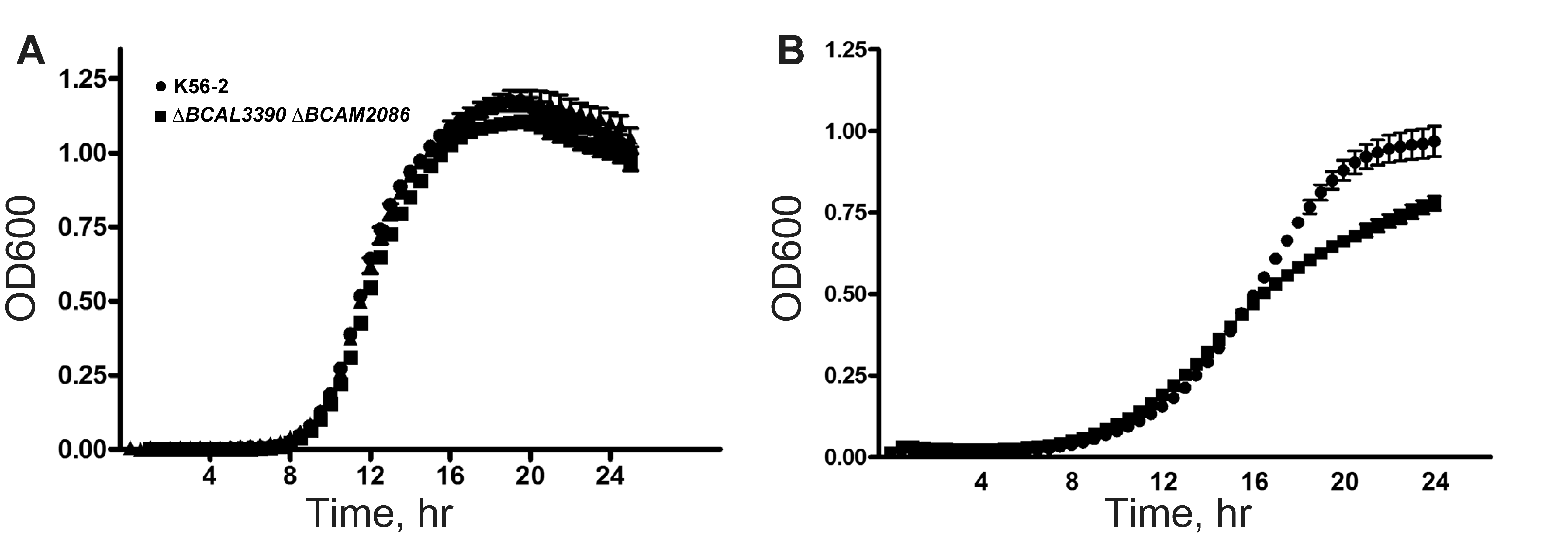

Supplement: Figure S5 — Effects of polyamine synthesis inhibitors on PmB resistance. The polyamine synthesis inhibitor dicyclohexylamine (blue) reduces the resistance of B. cenocepacia Δ rpoE/500 subpopulation to PmB, with no effect of 3-(methylthio)propylamine (red), shown in a turbidimetric PAP at 18 h; n = 5. (TIF) [file pone.0068874.s005.tif]

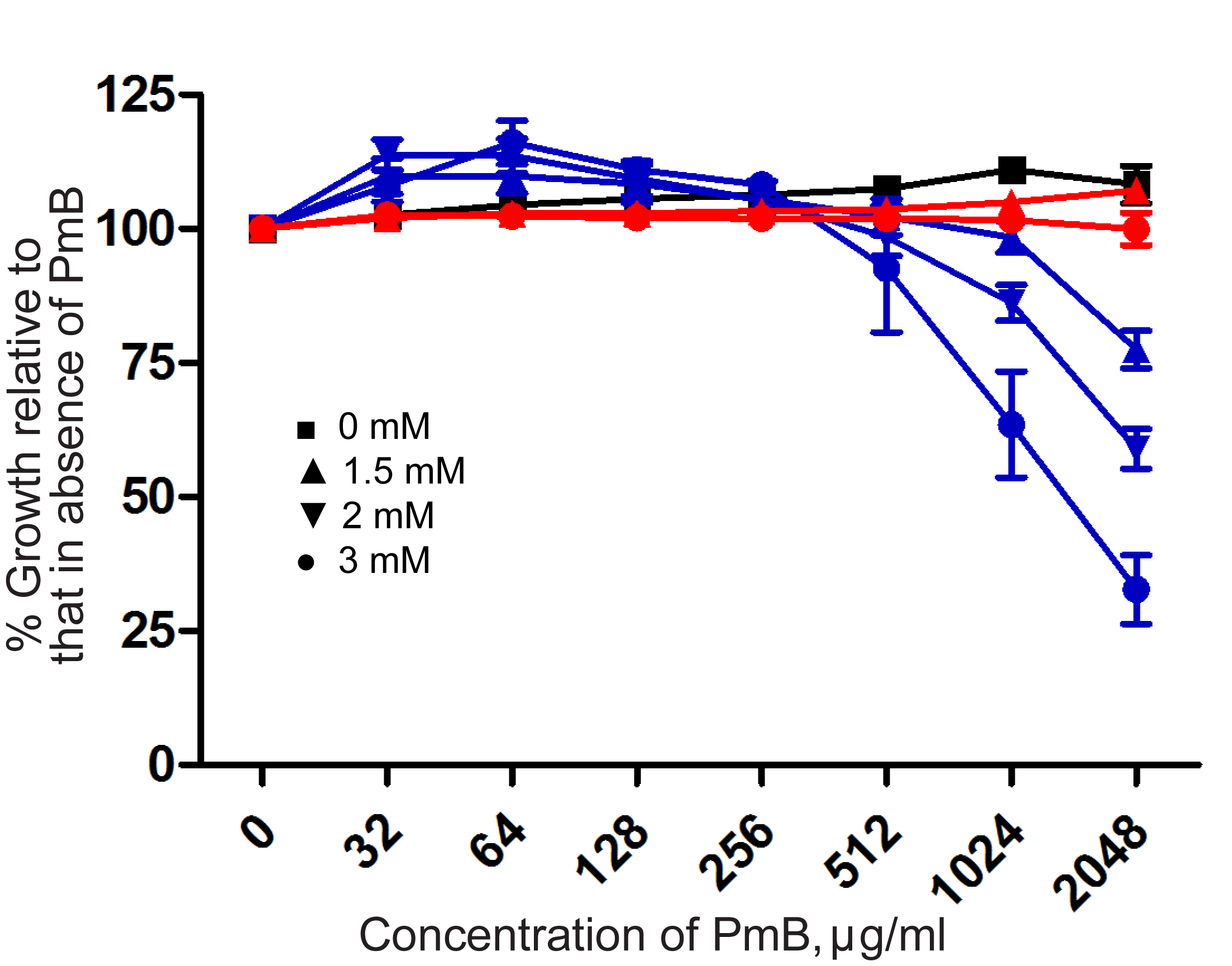

Supplement: Figure S6 — Ornithine decarboxylase (ODC) activity of B. cenocepacia K56-2 either untreated or treated with 1 mM of dicyclohexylamine or 3-(methylthio)propylamine at 24 h. This concentration of the polyamine synthesis inhibitors did not affect the growth of the bacteria. n = 9. (TIF) [file pone.0068874.s006.tif]

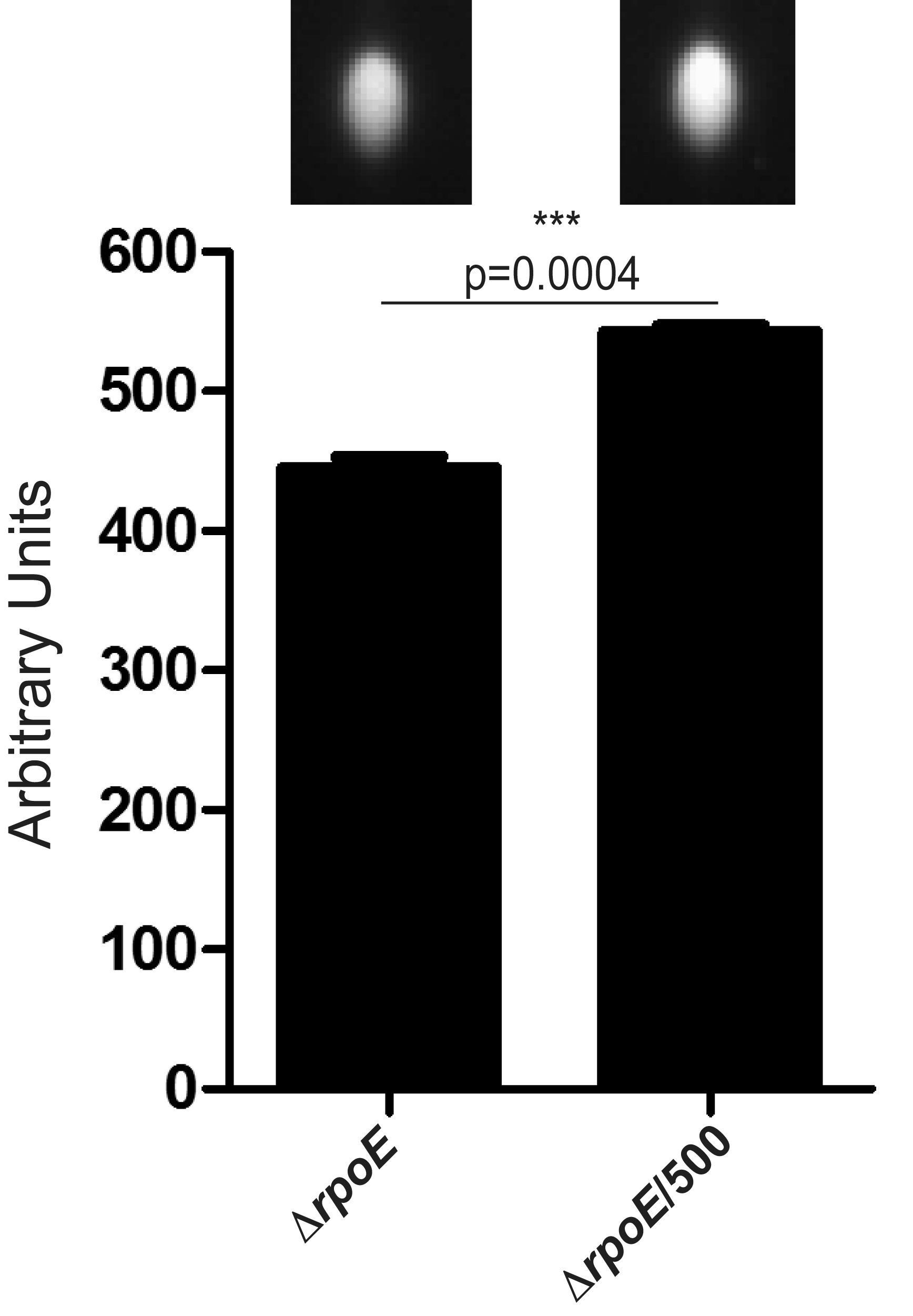

Supplement: Figure S7 — Release of putrescine in the Δ rpoE /500 culture supernantant. Increased release of putrescine in the supernatant of ΔrpoE/500 subpopulation treated with 500 µg/ml PmB relative to naïve ΔrpoE determined at 20 h from M9 cultures by TLC analysis. (TIF) [file pone.0068874.s007.tif]

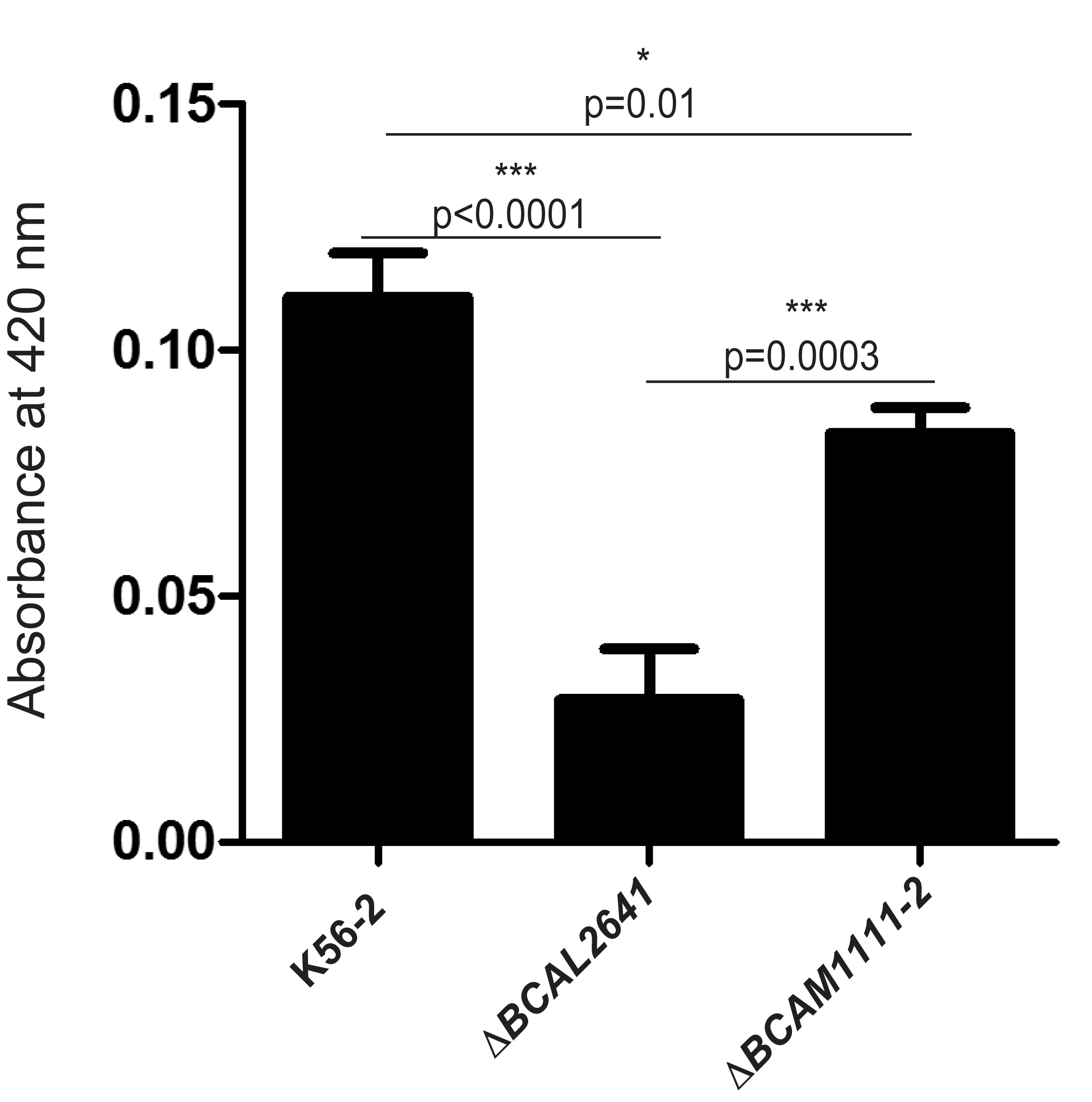

Supplement: Figure S8 — ODC assay of the parental strain K56-2 and different PAs biosynthetic mutants at 6 h. n = 9. (TIF) [file pone.0068874.s008.tif]
