# Supplementary material for: Chemical Communication of Antibiotic Resistance by a Highly Resistant Subpopulation of Bacterial Cells
Source: PLoS One. 2013 Jul 3;8(7):e68874. doi: 10.1371/journal.pone.0068874 (PMC3700957; doi:10.1371/journal.pone.0068874)
Supplement: Table S1 — MIC by agar dilution technique to determine the volatile-mediated protective effect of the supernatant of Δ rpoE /500 from the effects of PmB on sensitive bacteria. (PDF) [file pone.0068874.s009.pdf]

Table S1. MIC by agar dilution technique to determine the volatile-mediated protective effect of the supernatant of Δ*rpoE*/500 from the effects of PmB on sensitive bacteria.

	Sterile LB Control (n)	Supernatant of Δ*rpoE*/500 (n)
Sensitive bacteria	MIC, μg/ml	
B. cenocepacia K56-2Δ*arnBC*	0.25 (4)	0.5 (4)
E. coli DH5α	0.25 (4)	0.5 (4)
E. coli HB101	0.25 (3)/ 0.5 (1)	0.5 (4)
E. coli GT115	0.25 (4)	0.25 (4)
