# Supplementary material for: Chemical Communication of Antibiotic Resistance by a Highly Resistant Subpopulation of Bacterial Cells
Source: PLoS One. 2013 Jul 3;8(7):e68874. doi: 10.1371/journal.pone.0068874 (PMC3700957; doi:10.1371/journal.pone.0068874)
Supplement: Table S2 — Strains and Plasmids. (PDF) [file pone.0068874.s010.pdf]

Table S2. Strains and Plasmids

Strain or plasmid	Relevant characteristics[a]	Source and/or reference
Strains		
Burkholderia cenocepacia		
CP706-J	CF clinical isolate	Cleveland
K56-2	ET12 clone related to J2315, CF clinical Isolate	[b]BCRRC,[1]
Δ*cciI*	Deletion of *cciI* in K56-2	[2]
Δ*cepI*	Deletion of *cepI* in K56-2	[2]
Δ*rpfF*	Deletion of *rpfF* in K56-2	[2]
Δ*rpoE*	SAL65, Deletion of *rpoE* in K56-2	S. Loutet
Δ*hldA*	Deletion of *hldA* in K56-2	[3]
Δ*arnBC*	K56-2*Prha-arnT*Δ*arnBC*Δ*amrAB,* suppressor strain	[4]
Δ*BCAL3390*	OME2, Deletion of *BCAL3390* in K56-2	This study
Δ*BCAM2086*	OME3, Deletion of *BCAM2086* in K56-2	This study
Δ*yceI*	OME4, Deletion of *yceI*(*BCAL3310* and *BCAL3311*)in K56-2	This study
Δ*BCAM1679*	OME5, Deletion of *BCAM1679* in K56-2	This study
Δ*BCAL3390*Δ*BCAM2086*	OME7, Deletion of *BCAM2086* in K56-2 Δ*BCAL3390*	This study
Δ*BCAL1281*	OME8, Deletion of *BCAL1281* in K56-2	This study
Δ*BCAL2641*	OME11, Deletion of *BCAL2641* in K56-2	This study
Δ*BCAM1111*Δ*BCAM1112*	OME12, Deletion of *BCAM1111* and *BCAM1112* in K56-2	This study
K56-2 pSCrhaB2	OME19, K56-2 carrying pSCrhaB2, Tp^{R}	This study
Δ*yceI* pSCrhaB2	OME20, K56-2 Δ*yceI* carrying pSCrhaB2, Tp^{R}	This study
Δ*yceI*p*yceI*	OME21, K56-2 Δ*yceI* carrying *yceI* cloned into pSCrhaB2, Tp^{R}	This study
Δ*amrAB*	OME29, Deletion of *amrAB* in K56-2	This study
Δ*BCAL2641*Δ*amrAB*	OME30, Deletion of *amrAB* in K56-2Δ*BCAL2641*	This study
Δ*amrAB::BCAL2641*$^{+}$	OME31, Chromosomal *BCAL2641* integration in *amrAB* locus in K56-2Δ*BCAL2641*	This study
Escherichia coli		
DH5α	F^{-}ϕ80*lacZ* M15 *endA1 recA1 supE44 hsdR*17(r$_{K}^{-}$ m$_{K}^{+}$) *deoR thi-1 nupG supE44 gyrA96relA1 Δ*(*lacZYA-argF*)*U169*, λ–	Laboratory stock
GT115	F^{-} *mcrAΔ*(*mrr-hsdRMS-mcrBC*) ϕ80*ΔlacZΔ*M15 *ΔlacX74 recA1rpsL* (StrA) *endA1Δdcm uidA*(*Δ*MluI)::*pir-116 ΔsbcC-sbcD*	Invivogen, San Diego, CA
HB101	F^{-}*mcrBmrrhsd*S20(rB-mB-) *rec*A13 *leu*B6 *ara*-14 *proA2 lacY1 gal*K2*xyl*-5 *mtl*-1 *rpsL*20(Sm^{R}) *glnV*44 λ -	Laboratory Stock
SY327	*araD* Δ(*lac pro*) *argE*(*Am*) *recA56 rifr nalA*, λ *pir*	[5]
BL21	F^{-}*dcm ompT hsdS*(r$_{B}^{-}$m$_{B}^{-}$) *gal*	Novagen
Pseudomonas aeruginosa		
PAO1	Non-CF clinical isolate	[6]
Plasmids		
pDAI-SceI-SacB	*ori*$_{pBBR1}$, Tet^{R}, *P$_{dhfr}$*, *mob*$^{+}$, expressing I-SceI, SacB	[7]
pGPI-SceI	*ori*$_{R6K}$, ΩTp^{R} , *mob*$^{+}$, including an I-SceI restriction site	[3]
pMH447	pGPI-SceI derivative used for chromosomal complementation allowing gene integration in the gentamicin efflux pump	[4]
pRK2013	*ori*$_{colE1}$, RK2 derivative, Kan^{R}, *mob*$^{+}$, *tra*$^{+}$	[8]
pDel*BCAL3390*	pOE2, pGPI-SceI with fragments flanking *BCAL3390*	This study
pDel*BCAM2086*	pOE3, pGPI-SceI with fragments flanking *BCAM2086*	This study
pDel*yceI*	pOE4, pGPI-SceI with fragments flanking *BCAL3310* and *BCAL3311*	This study
pDel*BCAL1281*	pOE5, pGPI-SceI with fragments flanking *BCAL1281*	This study
pDel*BCAM1679*	pGPI-SceI with fragments flanking *BCAM1679*	[9]
pDel*BCAL2641*	pOE6, pGPI-SceI with fragments flanking *BCAL2641*	This study
pDel*BCAM1111*	pOE7, pGPI-SceI with fragments flanking *BCAM1111* and *BCAM112*	This study
pSCrhaB2	ori$_{pBBR1}$*rhaR*, *rhaS*, *P$_{rhaB}$*Tp^{R}*mob*$^{+}$	[10]
p*yceI*	pOE8, *yceI* cloned in pSCRha-B2	This study
p*BCAL2641*	pOE9, *BCAL2641* cloned in pMH447 for chromosomal complementation	This study
pET28a(+)		Novagen
pExp*BCAL3310*	pOE15, *BCAL3310* without signal peptide encoding sequence cloned in pET28a(+)	This study

pExp*BCAL3311*	pOE16, *BCAL3311* without signal peptide encoding sequence cloned in pET28a(+)	This study

[a]Tp^R, trimethoprim resistance, Kan^R, kanamycin resistance, Tet^R, tetracycline resistance.
[b]BCRRC, *B. cepacia* Research and Referral Repository for Canadian CF Clinics.

1. Mahenthiralingam E, Coenye T, Chung JW, Speert DP, Govan JR, et al. (2000) Diagnostically and experimentally useful panel of strains from the *Burkholderia cepacia* complex. J Clin Microbiol 38: 910-913.
2. Aubert DF, O'Grady EP, Hamad MA, Sokol PA, Valvano MA (2013) The *Burkholderia cenocepacia* sensor kinase hybrid AtsR is a global regulator modulating quorum-sensing signalling. Environ Microbiol 15: 372-385.
3. Flannagan RS, Linn T, Valvano MA (2008) A system for the construction of targeted unmarked gene deletions in the genus *Burkholderia*. Environ Microbiol 10: 1652-1660.
4. Hamad MA, Di Lorenzo F, Molinaro A, Valvano MA (2012) Aminoarabinose is essential for lipopolysaccharide export and intrinsic antimicrobial peptide resistance in *Burkholderia cenocepacia*. Mol Microbiol 85: 962-974.
5. Miller VL, Mekalanos JJ (1988) A novel suicide vector and its use in construction of insertion mutations: osmoregulation of outer membrane proteins and virulence determinants in *Vibrio cholerae* requires toxR. J Bacteriol 170: 2575-2583.
6. Holloway BW (1955) Genetic recombination in *Pseudomonas aeruginosa*. J Gen Microbiol 13: 572-581.
7. Hamad MA, Skeldon AM, Valvano MA (2010) Construction of aminoglycoside-sensitive *Burkholderia cenocepacia* strains for use in studies of intracellular bacteria with the gentamicin protection assay. Appl Environ Microbiol 76: 3170-3176.
8. Figurski DH, Helinski DR (1979) Replication of an origin-containing derivative of plasmid RK2 dependent on a plasmid function provided in trans. Proc Natl Acad Sci U S A 76: 1648-1652.
9. Tolman JS, Valvano MA (2012) Global changes in gene expression by the opportunistic pathogen *Burkholderia cenocepacia* in response to internalization by murine macrophages. BMC Genomics 13: 63.
10. Cardona ST, Valvano MA (2005) An expression vector containing a rhamnose-inducible promoter provides tightly regulated gene expression in *Burkholderia cenocepacia*. Plasmid 54: 219-228.
